# Retraction Note: The role of cyclooxygenase-2, interleukin-1β and fibroblast growth factor-2 in the activation of matrix metalloproteinase-1 in sheared-chondrocytes and articular cartilage

**DOI:** 10.1038/s41598-025-96380-1

**Published:** 2025-04-08

**Authors:** Pei-Pei Guan, Jing-Wen Guo, XinYu, Yue Wang, Tao Wang, Konstantinos Konstantopoulos, Zhan-You Wang, Pu Wang

**Affiliations:** 1 College of Life and Health Sciences, Northeastern University, Shenyang, 110819 People’s Republic of China; 2 Department of Chemical and Biomolecular Engineering, Baltimore, MD 21218 USA; 3 Johns Hopkins Institute for NanoBioTechnology, Baltimore, MD 21218 USA; 4 Johns Hopkins Physical Sciences-Oncology Center, Baltimore, MD 21218 USA; 5 Center of Cancer Nanotechonology Excellence, The Johns Hopkins University, Baltimore, MD 21218 USA

Retraction of: *Scientific Reports* 10.1038/srep10412, published online 20 May 2015

The Editors have retracted this Article.

A number of image integrity concerns were raised post-publication. Figure 3G appears to overlap with Fig. 3A of an earlier paper by the same authors, describing different conditions^1^. Within Fig. 2A, p-38 and β-actin blots appear to partially overlap. Figure 2C IL-1β and Fig. 5A 15d-PGJ_2_, and Fig. 6E WT appear to partially overlap with rotation. The Editors have lost confidence in the data and conclusions of this Article.

Authors Konstantinos Konstantopoulos agrees with this retraction. Author Wang Pu has not explicitly stated whether they agree or disagree with this retraction. All other authors did not respond to correspondence from the publisher regarding this retraction.
